# Analysis of College Students’ Personal Health Information Activities: Online Survey

**DOI:** 10.2196/jmir.9391

**Published:** 2018-04-20

**Authors:** Sujin Kim, Donghee Sinn, Sue Yeon Syn

**Affiliations:** ^1^ Division of Biomedical Informatics Department of Internal Medicine University of Kentucky Lexington, KY United States; ^2^ Department of Information Science University at Albany – State University of New York Albany, NY United States; ^3^ Department of Library and Information Science The Catholic University of America Washington, DC United States

**Keywords:** health records, personal, health information management, student health services

## Abstract

**Background:**

With abundant personal health information at hand, individuals are faced with a critical challenge in evaluating the informational value of health care records to keep useful information and discard that which is determined useless. Young, healthy college students who were previously dependents of adult parents or caregivers are less likely to be concerned with disease management. Personal health information management (PHIM) is a special case of personal information management (PIM) that is associated with multiple interactions among varying stakeholders and systems. However, there has been limited evidence to understand informational or behavioral underpinning of the college students’ PHIM activities, which can influence their health in general throughout their lifetime.

**Objective:**

This study aimed to investigate demographic and academic profiles of college students with relevance to PHIM activities. Next, we sought to construct major PHIM-related activity components and perceptions among college students. Finally, we sought to discover major factors predicting core PHIM activities among college students we sampled.

**Methods:**

A Web survey was administered to collect responses about PHIM behaviors and perceptions among college students from the University of Kentucky from January through March 2017. A total of 1408 college students were included in the analysis. PHIM perceptions, demographics, and academic variations were used as independent variables to predict diverse PHIM activities using a principal component analysis (PCA) and hierarchical regression analyses (SPSS v.24, IBM Corp, Armonk, NY, USA).

**Results:**

Majority of the participants were female (956/1408, 67.90%), and the age distribution of this population included an adequate representation of college students of all ages. The most preferred health information resources were family (612/1408, 43.47%), health care professionals (366/1408, 26.00%), friends (27/1408, 1.91%), and the internet (157/1408, 11.15%). Organizational or curatorial activities such as Arranging, Labeling, Categorizing, and Discarding were rated low (average=3.21, average=3.02, average=2.52, and average=2.42, respectively). The PCA results suggested 3 components from perception factors labeled as follows: Assistance (alpha=.85), Awareness (alpha=.716), and Difficulty (alpha=.558). Overall, the Demographics and Academics variables were not significant in predicting dependent variables such as Labeling, Categorizing, Health Education Materials, and Discarding, whereas they were significant for other outcome variables such as Sharing, Collecting, Knowing, Insurance Information, Using, and Owning.

**Conclusions:**

College years are a significant time for students to learn decision-making skills for maintaining information, a key aspect of health records, as well as for educators to provide appropriate educational and decision aids in the environment of learning as independent adults. Our study will contribute to better understand knowledge about specific skills and perceptions for college students’ practice of effective PHIM throughout their lives.

## Introduction

### Background

With abundant personal health information at hand, individuals are faced with a critical challenge in evaluating the informational value of the health care records to keep useful information and discard that which is determined useless. College students, in particular, are confronted with a similar issue; however, their situation is quite different from that of the senior population. Young, healthy college students who were previously dependents of adult parents or caregivers are less likely to be concerned with disease management. As such, their lack of interest in health care [1] leads to further disinterest in personal health document management. Personal health information management (PHIM) is a special case of personal information management (PIM) that is associated with multiple interactions among varying users (eg, patients, providers, insurance companies), complex health information and systems (eg, labs, medications, insurance), and advanced health information technology tools (eg, personal health records, PHRs; personal health devices) [2-4]. In PHIM research, little is known about college students’ information management activities in the context of health. Thus, this study investigates the demographic and academic profiles of college students with regard to diverse PHIM activities. Additionally, this study aims to discover the major determinants of key information management activities among college students for health information. This study reviews existing literature about diverse PHIM activities and document types and college students’ health information–seeking with relevance to their PIM behaviors.

### Personal Health Information Management Activities and Document Types

What individuals do with their personal health documents has been studied to understand diverse information management activities, document types, and related personal behaviors. As a health focus of PIM, core tasks of PIM or PHIM activities include “the search, retrieval, and re-finding of previously encountered information from both personal and shared space” [4,5]. Among these activities, individuals develop and use their own strategies to manage and organize their personal records. However, it is not clear if the strategies are effective or efficient. In the PIM context, researchers have observed that “the individual characteristic of being orderly has a positive bearing at a later point in time when the individual needs to find this information” [3,6-8]. Furthermore, successful PIM retrieval is dependent on the “prior processes used to organize relevant information and the extent to which those processes were appropriately planned” [3].

There is no comprehensive understanding of sources or document types contained in PHR systems. However, some PHIM studies reported specific or situational aspects of PHIM sources and document types. The most important PHR sources are health care providers who are broadly responsible for delivery and administration of health care. This group generates diverse types of health records (or documents) at clinical encounters such as care notes (eg, discharge summary, physical exam), therapeutic notes (eg, operative notes, treatment regimes, procedure information, surgeries), imaging or lab results (eg, x-rays, pathology, cytology), or administrative or legal or financial information (eg, appointment schedule, medical bills and receipts, birth certificate or death certificate, date of birth) [3]. Health care insurers were also reported as the relevant PHR source representing any health insurance program that “helps pay for medical expenses; whether through privately purchased insurance, social insurance or a social welfare program funded by the government” [3]. This information is then accessible for further investigation at times of inquiry or need.

Nowadays, some tethered PHRs can selectively or potentially include calendar or diary entries, daily planners, medications and tools, reference material, referrals, poison control, cancer surveys, over-the-counter medications, exercise and diet or self-care logs, home-monitored data (eg, blood pressure, glucose, peak flow), logs of symptoms, or pedometer data [9-11]. Additionally, individuals’ social networks such as family, friends, and other informal human sources were reported as relevant PHR sources. In recent times, online support communities of people with similar diseases, such as PatientsLikeMe, also constitute relevant PHR sources [12]. Traditional public health sources such as the mass media, public health departments, and libraries still play important roles as PHR sources through health websites, printed health publications, public library classes, etc.

### Personal Information Management Perceptions

Although the previous studies have not focused on college population for their health documents, they have identified some interesting findings regarding factors influencing PIM activities [13,14]. Technological solutions or individual knowledge about diverse PIM tools and methods were found to be associated with individuals’ success at PIM management [14], especially in digital environments [13,14], as individual users often have limited knowledge about appropriate technical tools or techniques for management and preservation [15]. As PIM technology evolves, diverse PIM activities happen in digital forms, and personal data stores could be at risk in terms of digital service providers’ policies and standards [16]. Williams et al reported that technologically perceptive interviewees were diligent with aspects concerning back-ups and mindful about the risk of loss, which was also confirmed in the research of Sinn et al [17,18]. Still, how technology influences young college students in the digital generation in terms of PHIM remains unknown.

The difficulties in PIM activities were investigated, and the 2 most critical challenges that were identified were curatorial and organizational activities. Bruce and colleagues (2005) found that individuals have difficulty in determining the future value of digital content [19]. Marshall similarly described curatorial decisions as a “cognitively demanding exercise” [13]. Individuals’ management methods for information are much more diverse in personal settings than those in organizational settings. People often allow their information to accumulate without taking action to organize it. Actions for decluttering or organizing personal information often happens with trigger events, such as moving offices, buying new devices, and reaching the limits of space capacity [17]. Even in cases where individuals preserve their content, their organizational activities for long-term use seem unlikely to meet a required level, and they especially lack “creating appropriate metadata, and migrating materials to maintainable formats” in a proper and secure data management system [16,20].

To achieve a satisfying level of information retrieval for individuals’ needs, some types of assistance, whether technological or professional, might be useful. The patterns of individuals’ preservation seem haphazard or premature, such as simple replications or keeping everything including old computers [13,15,17,21-23]. Obviously, those patterns are neither sustainable nor efficient. Many researchers argue that professional intervention in PIM would benefit them greatly to preserve important personal information as well as to preserve cultural heritage from which personal histories could be found [14,17,18]. However, the era of professional support or technological assistance in PIM is still in its infancy, with only limited technological support available mainly for the aging population [24,25].

The sense of ownership or home-grown organization was one of the ways to observe the characteristics of a personal archive [26]. The same applies to the online environment. For example, users perceive the Cloud as a “storage box” on the internet, not going much beyond the concept of ownership [27]. In addition, individuals strongly felt that they should be able to preserve even their own social media data [28]. Hence, the sense of possession or ownership may influence PIM activities.

Another factor was awareness of the importance of personal information. When someone thinks that his or her personal information may be important in a different context (eg, financial, academic, personal history), then he or she may make more of an effort to preserve that information. Personal information builds personal life history, documents important occasions for achievements or memorials, and presents identity construction evidence [26,29]. Although proven to be associated with PIM activity [18], the awareness, however, has not been tested for any specific context, such as health information, college students, or other PIM activities.

### College Students’ Health Information–Seeking Behaviors

College students enter a critical transition and begin to become independent and responsible for their own health during college years. As they are away from their parents, college students must acquire their health records, such as immunization records, drug test results, or vaccination records, and present them whenever asked for academic admission or employment. Moreover, college students are thought to be a vulnerable population in that they are exposed to pandemic outbreaks such as meningococcal disease and influenza [30-32]. However, they often exhibit lack of interest in either disease management or health information management. Most importantly, this age group is the least insured in the United States [32], in spite of the fact that they are exposed to risky behaviors, such as the highest rates of motor vehicle injury and death, homicide, mental health problems, sexually transmitted infections, and substance abuse [31,32]. In addition, these young adults do not normally seek assistance with finding or maintaining their PHRs until an illness or accident occurs [30].

Studies have reported that college students are using online resources for health information due to their easy access, although the students do not consider them to be credible [33-36]. Given that health and medical information requires professional knowledge to interpret and manage [35,36], this situation could lead to critical health decisions. In this sense, the fact that personal health record keeping has not been a part of college education in a conventional academic setting is problematic. Particularly for health matters, having unknown digital records that hold important personal information may mean being at an increased risk of chronic conditions and their associated complications for many more years, thus making college students an important population in need of immediate health promotion and intervention.

With relevance to health information seeking and sharing activities, Syn and Kim (2016) reported that both contextual and user variations were influencing factors [37]. Ivanitskaya and colleagues reported that “most students (89%) understood that a one-keyword search is likely to return too many documents,” and that “few students were able to narrow a search,” showing search inefficacy among college students [38]. They also reported that “students’ self-perceptions of skill tended to increase with increasing level of education” [38]. Notably, as part of Project Information Literacy, Head and Eisenberg (2009) reported that college students in their survey “used course readings and Google first for course-related research and Google and Wikipedia for everyday life research” [39]. As such, there has been limited evidence to understand informational or behavioral underpinning of the college students’ PHIM activities, which can influence their health, in general, throughout their lifetime. This knowledge can help students practice effective PHIM throughout their lives.

Therefore, the aim of this study was to investigate perceived behaviors of college students by asking questions that focused on various information management–related activities through an online survey. Three research questions were tested within our samples. The first research question investigated demographic and academic profiles of college students with relevance to PHIM activities. The second research question sought to construct major PHIM-related activity components and perceptions among college students. The third research question sought to discover the major factors predicting core PHIM activities among college students that we sampled.

## Methods

### Survey Sample

The target sample was 28,254 students who were included on the University of Kentucky student mailing list (as of January 2017). We excluded those who signed off from the University mailing list according to the Family Educational Rights and Privacy Act. Our online survey responses were collected from March through May 2017. The overall response rate was 9.12% (2578/28,254), and the study included only responses with a survey completion rate greater than 90% (1408/2578, 54.61%). The participants who included their emails participated in a drawing for compensation. The study has been approved by the University of Kentucky institutional review board.

### Nonresponse Analysis and Common Method Bias

Low response rate for Web surveys among college students is not a surprising phenomenon. As reported in the recent National Survey of Student Engagement, the response rate ranged from 5% to 77%, with an average of 29% [40]. Our data show a high dropout rate of 44.57% (1149/2578) where the remaining responses were completely missing. Due to low response rate (ie, 9.12%), nonresponse analysis recommended by Babbie (1990) was performed by comparing the initial 30% and the last 30% responses (considered as a proxy for nonresponses) [41]. To compare the 2 groups, we performed the analysis of variance test, which indicates no statistically significant differences between the 2 groups of respondents for the independent and dependent variables. For instance, the demographics variables entered in hierarchical regression analyses, age (*F*_1,784_=2.532, *P*=.11 gender (*F*_1,785_=0.588, *P*=.44), ethnicity (*F*_1,784_=0.849, *P*=.36), and relationship (*F*_1,788_=0.247, *P*=.62). Remaining variables entered in the regression analyses were found to be insignificant between the 2 groups. Therefore, the nonresponse bias is considered to be minimal in this study.

Additionally, Harman single-factor test based on confirmatory factor analysis was performed to avoid the common method bias [42]. This study employed the online survey method to measure college students’ information management behaviors and perceptions with relevance to personal health record management within the same survey respondents. Therefore, the common method bias issue can be introduced by measuring both independent and dependent variables that were collected from the same survey respondents. Harman single-factor test shows that the largest variance for one factor (ie, age) is 12.92%, which is less than the acceptable value of 50% [43]. Therefore, the common method bias is also considered to be marginal in this study.

### Measures

An aggregate sum of 84 PHIM-related activities measuring from 1 (strongly disagree) to 5 (strongly agree) on the Likert scale for each question was used as a dependent variable, namely, Overall PHIM Activity. These 84 questions used as a PHIM activity measure were based on literature [3-8] in our reference. In addition to the overall PHIM score, we formed 11 additional dependent variables using a principal component analysis (PCA) using SPSS v.24. The PCA allows us to convert possibly correlated variables into principle components by the strengths of possible variances, so that we could create principle PHIM activities and record type constructs. The surveyed items and accompanying results are reported in Tables 1-3. From the PCA analysis, the 11 PHIM constructs formed include Labeling, Sharing, Categorizing, Collecting, Health Education Materials, Understanding, Discarding, Insurance Information, Organizing, Using, and Owning. Reliability scores of Cronbach alpha for these 11 PHIM components range from .803 to .969, indicating high internal consistency in PHIM measures (Table 4). For predictor variables, 16 survey questions were analyzed to extract major PIM perception components using the PCA technique. We included only highly reliable components among our predictors for a series of regression analyses. The PIM perceptions used as predictors are labeled as Assistance, Awareness, and Difficulty (Table 3). Additionally, demographics (age, gender, ethnicity), health concerns (number of clinic visits, preferred health information resources), and academic characteristics (status, relationship, grade point average, number of courses taken) were entered in hierarchical regression analyses to predict major predictors for the PHIM activities extracted. To control demographics or academic variances, we recoded some variables into a binary comparison (Female: 1, others: 0; White: 1, others: 0; Undergraduate: 1, others: 0; Single: 1, others: 0) in our hierarchical regression analyses. The preferred health information sources were measured from 1 (representing least preferred) to 9 (most preferred).

## Results

We first performed a descriptive analysis to characterize our student sample, which was followed by PCA analyses. On the basis of the PCA results, a series of hierarchical regression analyses were performed to assess which variables were statistically meaningful to predict diverse PHIM activities.

### Research Question 1: Sample Characteristics

The first research question sought to profile demographic characteristics of the survey participants (N=1408; Table 1). The majority of participants were female (956/1408, 67.90%), and the age distribution of this population included adequate representation of college students of all ages, except for adults aged 66 years or above (n=8). The participants were oversampled in female population in comparison with University of Kentucky’s current student demographics (N=16,628; 54.10%). This sample lacked racial and ethnic diversity in that 72.70% (1023/1408) were white, with African Americans representing the next most sampled population (5.90%, 83/1408). For academic status, 852 students (852/1408, 60.50%) were undergraduate, and the rest represented graduate or certification program students. Half of the students resided in off-campus housing (824/1408, 58.50%), and 48.90% (689/1408) reported being in a relationship. High grade averages were reported, with A (800/1408, 56.80%), B (413/1408, 29.30%), C (87/1408, 6.20%), D (9/1408, 0.60%), and F (9/1408, 0.60%), Participants reported that they predominantly use parent-provided health insurance (787/1408, 55.90%), the student health plan through the University (206/1408, 14.60%), and employment-based insurance (177/1408, 12.60%).

**Table 1:** Sample description—demographics, academics, health, and information resources (N=1408).

Variables		Statistics
Age, mean (SD)		24.65 (7.10)
**Gender, n (%)**		
	Male	354 (25.14)
	Female	956 (67.90)
	No response	98 (7.00)
**Ethnicity, n (%)**		
	White, not Hispanic	1023 (72.70)
	Black, not Hispanic	83 (5.90)
	Hispanic or Latino	53 (3.80)
	Asian or Pacific Islander	106 (7.50)
	Native American or Alaskan Native	3 (0.20)
	Other	44 (3.10)
	No response	96 (6.80)
**Academic status, n (%)**		
	1st year undergraduate	237 (16.80)
	2nd year undergraduate	197 (14.00)
	3rd year undergraduate	189 (13.40)
	4th year undergraduate	173 (12.30)
	5th year or more undergraduate	56 (4.00)
	Mater’s program	154 (10.90)
	Doctoral program	267 (19.00)
	Certification program	5 (0.40)
	Other: please specify	36 (2.60)
	No response	94 (6.70)
**International students, n (%)**		
	Yes	86 (6.10)
	No	1227 (87.10)
	No response	95 (6.70)
**Housing, n (%)**		
	Campus residence hall	315 (22.40)
	Fraternity or sorority house	30 (2.10)
	Other university housing	36 (2.60)
	Off-campus housing	824 (58.50)
	Parent or guardian’s home	71 (5.00)
	Other: please specify	38 (2.70)
	No response	94 (6.70)
**Relationship, n (%)**		
	Single	689 (48.90)
	Married/domestic partner	188 (13.40)
	Engaged/committed dating relationship	403 (28.60)
	Separated	8 (0.60)
	Divorced	13 (0.90)
	Widowed	2 (0.10)
	Other: please specify	13 (0.90)
	No response	92 (6.50)
**Tuition support, n (%)**		
	Parents	521 (37.00)
	Student loans	509 (36.20)
	Self	441 (31.30)
	Your employer	94 (6.70)
	Scholarships (eg, teaching/research assistantship)	683 (48.50)
**Grade point average, n (%)**		
	A	800 (56.80)
	B	413 (29.30)
	C	87 (6.20)
	D/F	9 (0.60)
	No response	99 (7.00)
**Health insurance, n (%)**		
	Parent health insurance	787 (55.90)
	Employment-based insurance	177 (12.60)
	Student health plan through universities	206 (14.60)
	Subsidized Obamacare coverage	17 (1.20)
	Catastrophic coverage	2 (0.10)
	Medicaid	68 (4.80)
	Not insured	23 (1.60)
	Other: please specify	24 (1.70)
	No response	104 (7.40)
**Health information sources sought first, n (%)**		
	Professionals (eg, doctors, nurses, etc.)	366 (26.00)
	Family	612 (43.50)
	Friends	27 (1.90)
	Colleagues (eg, other patients)	6 (0.40)
	Internet	157 (11.20)
	Social media	2 (0.10)
	Mass media	1 (0.10)
	Government agencies	3 (0.20)
	Libraries	3 (0.20)
	Other	16 (1.10)
Number of courses taken, mean (SD)		27.08 (22.64)
Number of clinic visit, mean (SD)		4.32 (6.33)
Number of digital devices owned, mean (SD)		3.18 (1.93)
Number of mobile phones owned, mean (SD)		3.57 (11.94)

**Table 2:** Personal health information management (PHIM) activities by document types.

PHIM activities by document types	Immunization records, mean (SD)	Family medical history, mean (SD)	Emergency information, mean (SD)	Surgery, mean (SD)	Drugs, mean (SD)	Insurance information, mean (SD)	Health education materials, mean (SD)	Average activities
I already have a collection of________	3.46 (1.28)	2.99 (1.23)	3.89 (1.12)	3.42 (1.26)	3.71 (1.19)	4.15 (0.95)	2.95 (1.30)	3.51
I have a habit of collecting ________ whenever providing for my health	3.27 (1.32)	3.04 (1.25)	3.56 (1.24)	3.28 (1.29)	3.52 (1.26)	3.81 (1.18)	2.91 (1.31)	3.34
I know which of ________ are needed for my doctor’s visits	3.55 (1.28)	3.65 (1.21)	3.95 (1.10)	3.71 (1.21)	3.95 (1.11)	4.09 (1.04)	3.29 (1.31)	3.74
I discard ________ when they are no longer needed	2.2 (1.10)	2.2 (1.08)	2.21 (1.12)	2.21 (1.11)	2.53 (1.26)	2.49 (1.28)	3.08 (1.38)	2.42
I have my own method to manage and organize	3.24 (1.30)	3.14 (1.29)	3.41 (1.26)	3.23 (1.29)	3.4 (1.26)	3.6 (1.21)	3.01 (1.30)	3.29
I categorize ________ on a regular basis	2.47 (1.21)	2.41 (1.17)	2.58 (1.24)	2.45 (1.19)	2.66 (1.26)	2.72 (1.28)	2.36 (1.16)	2.52
I arrange ________ effectively so that I can find it easily for my doctor’s appointment	3.15 (1.35)	3.03 (1.31)	3.35 (1.32)	3.09 (1.32)	3.37 (1.31)	3.66 (1.24)	2.79 (1.32)	3.21
I label ________ in a meaningful way so I can find it easily for later use for my doctor’s appointment	3.02 (1.37)	2.9 (1.33)	3.12 (1.36)	2.97 (1.34)	3.16 (1.35)	3.3 (1.36)	2.7 (1.30)	3.02
Usually, I try to personally own a copy of ________ in my possession	3.33 (1.36)	3.02 (1.32)	3.47 (1.32)	3.09 (1.33)	3.47 (1.31)	3.96 (1.15)	2.75 (1.33)	3.30
I can easily find ________ in an efficient manner	3.39 (1.31)	3.28 (1.29)	3.76 (1.20)	3.37 (1.29)	3.69 (1.21)	3.98 (1.08)	3.07 (1.34)	3.51
I can easily share my________ records, when needed	3.36 (1.31)	3.28 (1.27)	3.74 (1.21)	3.42 (1.27)	3.67 (1.23)	3.91 (1.10)	3.01 (1.32)	3.48
I use ________ when I discuss my health matters with a health professional	3.24 (1.27)	3.59 (1.18)	3.69 (1.16)	3.57 (1.21)	3.89 (1.10)	3.86 (1.11)	2.94 (1.30)	3.39
Average by data types	3.14	3.04	3.39	3.15	3.42	3.63	2.91	

The most preferred health information resources were as follows: family (43.50%, 612/1408), health care professionals (366/1408, 26.00%), friends (27/1408, 1.90%), and the internet (157/1408, 11.20%). Compared with other studies, this sample prefers depending more on family for health information sources than health care professionals [1,33]. Although this is not a direct comparison, the average number of clinic visits in this sample was 4.32 times more than those in the past year, implying a relatively healthy population compared with the national average of 12.9 visits in 2001 and 11.6 visits in 2010 among people aged between 18 and 64 years who reported fair or poor health [44].

Among the 12 PHIM activities, the participants reported that “I know which of (document types) are needed for my doctor’s visits” (average=3.74) ranked the highest (Table 2). Organizational or curatorial activities such as Arranging, Labeling, Categorizing, and Discarding were rated low (average=3.21, average=3.02, average=2.52, and average=2.42, respectively). For the record types–related questions, we found that Insurance information was the PHIM data type that was most actively managed (average=3.63), whereas Health Education Materials and Family Medical Histories were the least favorably pursued PHIM data types (average=2.91 and 3.04, respectively).

### Research Question 2: Major Personal Health Information Management Constructs

The second research question sought to identify principle factors for PHIM activities using PCA analyses. In addition to the demographic information, we included PIM perception factors as predictors. The PCA results suggested 5 components from perception factors, 2 of which were eliminated due to low reliability scores, resulting in 3 components labeled as follows: Assistance (alpha=.85), Awareness (alpha=.716), and Difficulty (alpha=.558). Table 3 reports further details on PCA results performed on PIM perceptions.

**Table 3:** Primary factors of personal information management (PIM) perceptions.

Components		Factor 1	Factor 2	Factor 3
**Factor 1: Assistance**				
	If I have professional assistance, I think I will be able to manage my personal records better	0.812		
	I would like professional advice about managing personal records	0.844		
	Training would be useful to manage my personal records better	0.848		
	I would like to have technology assistance to manage my personal records	0.738		
**Factor 2: Awareness**				
	It is important to keep my personal records for future use		0.812	
	It is critical to collect my academic records for my future career		0.764	
	It is essential to store my health records to better manage my health		0.707	
**Factor 3: Difficulty**				
	It takes considerable time to look through my personal records to determine what to keep and what to delete			0.830
	I find it difficult to know how I should organize my personal records			0.659
Mean (SD)		3.300 (1.051)	4.220 (0.782)	3.370 (1.093)
Cronbach alpha		.85	.716	.558
Eigenvalue		3.607	2.882	1.003
Percentage of variance explained		22.542	18.013	6.271

**Table 4:** Summary of PCA results by component for primary factors in personal health information management (PHIM) activities for record types.

PCA Result Summary by Components	1	2	3	4	5	6	7	8	9	10	11
Cronbach alpha	.969	.933	.971	.924	.895	.944	.925	.803	.964	.922	.933
Mean	3.052	3.550	2.500	3.335	2.962	3.795	2.414	3.933	3.250	3.692	3.252
SD	1.347	1.244	1.205	1.270	1.305	1.166	1.179	1.113	1.289	1.162	1.329
Eigenvalue	40.867	6.606	4.502	2.901	2.415	2.102	2.050	1.727	1.655	1.508	1.420
Percentage of variance explained	48.651	7.865	5.359	3.454	2.875	2.503	2.440	2.056	1.970	1.795	1.690

On the basis of the responses to 84 PHIM questions, we performed a PCA analysis to form statistically meaningful constructs for use as dependent variables in the hierarchical regression analyses. As a result, the model yielded 11 distinct factors that represent 11 PHIM activities (Multimedia Appendix 1 and Table 4). The factors accounted for about 78.9% of the variance. The scores for the scales were summed and divided by the number of items in the scale to produce variables ranging from 1 to 5, with smaller values indicating lower levels of agreement. The reliability of the 11 factors was also assessed to measure strengths of the scales. The 11 factors were subsequently labeled as follows: Labeling (alpha=.969), Sharing (alpha=.933), Categorizing (alpha=.971), Collecting (alpha=.924), Health Education Materials (alpha=.895), Knowing (alpha=.944), Discarding (alpha=.925), Insurance Information (alpha=.803), Organizing (alpha=.964), Using (alpha=.922), and Owning (alpha=.933). Multimedia Appendix 1 reports the full PCA result.

### Research Question 3: Predicting Primary Factors for Personal Health Information Management Activities

The last research question sought to discover which independent variables are affecting factors to the major PHIM activities constructed from the PCA. The relationship between possible factors from the college students’ characteristics and the 12 PHIM activity constructs is the focus of the third research question. A series of 12 dependent variables (overall PHIM activity + 11 PHIM activity constructs) were tested with regression analyses using 4 groups of factor variables, including Demographics, Academics, Health Resources, and PIM Perceptions. For the hierarchical regression analyses, Demographics variables were entered in the first block, Academics variables—GPA, number of courses taken, academic status—were entered in the second block, Health and Information Resources variables—number of clinic visits and the 5 health information sources including professionals, family, friends, the internet, and mass media—were entered in the third block, and 3 PIM Perceptions variables of assistance, awareness, and difficulty were entered in the fourth block. Multimedia Appendix 2 shows an aggregate result of the hierarchical regression analyses between the 4 independent variables (predictors) and the 12 dependent variables (PHIM activities), and Table 5 shows the hierarchical regression analysis predicting overall personal health information management (PHIM) activity.

Overall, Health and Information Resources and Perceptions significantly increased the explanatory power of the regression model. More specifically, the Demographics and Academics variables were not significant in predicting the dependent variables such as Labeling, Categorizing, Health Education Materials, and Discarding, whereas they were significant for other outcome variables. Among Demographics variables, gender (coded female=1) significantly explained the outcome variables of Sharing, Collecting, Knowing, Insurance Information, Using, and Owning. Although the overall Academics variables significantly explain some activities, none of the individual Academics variables are significant for each dependent construct. For Health and Information Resources variables, the number of clinic visits is significant in Sharing and Using variables. Some of preferred health information resources such as professionals and friends are found to be significant factors in some PHIM activity variables. The internet and mass media did not significantly predict most of the PHIM variables. Among the 3 PIM Perceptions variables, Awareness is the most significant of the 12 outcome variables, whereas the Difficulty variable is not significant in health education–related and discarding activity. Interestingly, the Assistance variable is found to be significant in Labeling, Sharing, and Organizing variables.

A 4-stage hierarchical multiple regression was conducted with the overall PHIM activity construct as a dependent variable for predictor variables used in the below analysis.

**Table 5:** Hierarchical regression analysis predicting overall personal health information management (PHIM) activity.

Dependent predictors		Overall personal health information management (PHIM) activity							
		*R*	*R* ^2^	Δ *R*^2^	Δ *F*	Degrees of freedom	Β	*t*	*P* value
**Demographics**		.139	.019	.019	5.272	4,1069		5.272	<.001
	Age						0.532	1.360	.17
	Gender^a^ (Female)						13.392	2.885	.004^b^
	Ethnicity^c^ (White)						4.745	0.945	.35
	Relationship^d^ (single)						−5.940	−1.393	.16
**Academics**		.148	.022	.003	0.979	7,1066		3.432	<.001
	Grade point average						3.594	1.082	.28
	Number of courses taken						0.028	0.312	.76
	Academic^e^ status						7.863	1.406	.16
**Health and information resources^f^**		.262	.069	.047	8.872	13,1060		6.025	<.001
	Number of clinic visit						0.626	1.964	.05^b^
	Professional						5.271	3.467	<.001
	Family						0.755	0.444	.66
	Colleague						3.993	2.614	.009^b^
	Internet						−1.931	−1.235	.22
	Mass media						−2.496	−1.314	.19
**Personal information management (PIM) perceptions^g^**		.379	.144	.075	30.874	16,1057		11.098	<.001
	Assistance						−1.445	−2.215	.03^b^
	Awareness						9.506	8.589	<.001
	Difficulty						−4.216	−3.431	<.001

^a^Gender: Dummy-coded with Female=1 and Male=0.

^b^*P*<.05.

^c^Ethnicity: Dummy-coded with White=1 and Others=0.

^d^Relationship: Dummy-coded with Single=1 and Others=0.

^e^Status: Dummy-coded with Undergraduates=1 and Others=0.

^f^Preferred Health Information Resources: 1 (least preferred) to 9 (Most preferred).

^g^PIM Perceptions: five-point response scale from Strongly Disagree (1) to Strongly Agree (5).

The hierarchical multiple regression revealed that at stage one, Demographics variability as a group contributed significantly to the regression model, *F*_4,1069_=5.272, *P*<.001, and accounted for 1.9% of the variation in overall PHIM variables. However, when considering the individual variables, only gender variation indicates significant contribution, whereas the other Demographic variables are not significant. Introducing the Academics variables at the next stage does not explain any additional variation in overall PHIM activity, but this change in *R*^2^ is significant statistically at *F*_7,1066_=3.432, *P*<.001. Adding Health and Information Resources variables to the regression model explains an additional 4.7% of the variation in overall PHIM activity, and this change in *R*^2^ is significant by *F*_13,1060_=6.025, *P*<.001. At the final stage, the addition of PIM Perceptions to the regression model explains an additional 7.5% of the variation in overall PHIM activity, and this change in *R* ² is also significant by *F*_16,1057_=11.098, *P*<.001. When all the 4 blocks of independent variables were included in stage 4 of the regression model, Assistance, Awareness, and Difficulty perceptions were found to be significant. Female, Professional, and Mass Media Resources variables were also found to be significant in predicting overall PHIM activities. The most important predictor of overall PHIM activity is the PIM Perceptions predictor, which uniquely explains 7.5% of the variation in overall PHIM activity. Taken together, all the 5 independent variables accounted for 14.4% of the variance in overall PHIM activity.

## Discussion

### Overall Characteristics

This study examined college students’ behaviors and perceptions about information management of their health-related information. Demographically, our sample is young, female, single, and white dominant, and this study sample is similar to that of the University of Kentucky student body, except for oversampled female students. These young adults reported high GPAs and generally seek health information from family and health professionals. Their total number of annual clinic visits indicates a relatively healthy population compared with the national average. The most active PHIM behaviors found were to know the value of health records, and collect and easily find the records. Their least active PHIM behaviors were to discard health information when no longer needed, and to categorize and label them for proactively organizing them. They highly value insurance and drug information but consider health education materials and family medical history less important for PHIM activities.

### Descriptive Characteristics

The descriptive analysis suggests that our study participants are heavily dependent on parents in terms of their financial support, including tuition payments and health insurance. It is not surprising, then, that the majority of college students are transitioning to build financial as well as medical independence from their parents. Their financial responsibilities speak not only to monetary dependency but also to information dependency on their parents or family members. In terms of information sources they use, we anticipate that authentic information sources such as clinicians or other health care providers would be sought by the students. However, majority of the participants report that they sought health information through family members first. This result is not consistent with other studies reporting that health care providers are initially or most frequently sought out as a health information source [1,25,36], and it is tied to our previous discussion about college students’ transitioning phase of information dependency.

### Affecting Factors

Our PCA analyses suggested 3 perception constructs, namely, Assistance, Awareness, and Difficulty. Among these, Assistance represents any professional or technological help in managing various PHIM activities, which have been discussed in previous research. Conversely, this study found that neither Technology nor Ownership questions were formed as significant factors, so we did not include them in our regression analyses. Surprisingly, we found that PIM Perceptions overall were a predictor construct, as well as the individual variables of Assistance, Awareness, and Difficulty. Additionally, Awareness and Difficulty were formed as statistically viable constructs for further analyses. Awareness indicates how college students perceive the importance of information management in their personal records and was shown to be highly perceived in this group. Thus, we entered this variable in our regression analyses.

As dependent variables, we formed 11 constructs out of 84 questions. Interestingly, the constructs formed such as Collecting, Organizing, or Using were consistent with previous PHIM literature [3,25]. However, some of the major PHIM activities such as Retrieving, which were heavily studied in information retrieval or seeking studies, were not statistically significant in internal consistency to form a construct and thus not included in our analyses. Although the descriptive values were not high, some variables, such as Labeling and Categorizing, presented good patterns as components. Curatorial activities such as Categorizing, Labeling, or Organizing were also important constructs in our PCA result. Among health information types, Health Education Materials and Insurance Information were used as outcome constructs in this analysis, even though their descriptive values were low. This result calls for further study to evaluate critical values of information contained in these underutilized resources.

The most interesting result was based on a series of 12 hierarchical regression analyses, as these provided an opportunity to examine the aforementioned predictors and their influence on diverse PHIM activities. With these regression results, we could identify possible factors for further analysis to better prepare in PHIM. For instance, many studies have examined the challenges that individuals experience during digital archiving practices [14,17,19,20,23,28,45]. As we stated, our goal was to identify a profile for active PHIM performers and to discover possible improvements based on the current state of PHIM practices and perceptions among college students. The overall results in Table 5 confirm that Demographics (Gender), Academics in general, Health Information Sources (number of clinic visits, professionals and colleagues as Information Sources), and PIM Perceptions (Assistance, Awareness, and Difficulty) have meaningful influences on PHIM variables. Therefore, we can profile active PHIM performers as female students with good academic standing, who visit clinics frequently and seek information from professionals and colleagues, who are aware of the importance of personal history, who acknowledge the importance of professional assistance, and who understand the difficulties of information management activities.

Females were the dominant population of our study group, and other PIM or information-seeking studies have already reported that females are active information seekers who were found to be better organizers than their counterparts [1,24]. In the second block, we found significant association between overall academic variances and PHIM activities. Academic status—undergraduate versus graduate—was found to be an insignificant predictor of certain PHIM activities such as Collecting or Sharing. In the third block, the number of clinic visits was an important predictor for Sharing and Using, and some health information sources such as professional and colleagues were found to be significant predictors. These 2 sources were found to be highly ranked health information sources and significant predictors of some curatorial PHIM activities including Labeling, Sharing, Categorizing, and Collecting. Such curatorial activities for deciding what to keep and what to delete were reported as the least performed PIM activity for a number of reasons [19]. For instance, whether a record has specific values for future use was difficult to judge for those who have no experience in PIM practices. Even if individuals have PIM experiences, anticipating changing PHIM status is a moving target that any human could adjust depending on unpredictable health conditions. This so-called “post-value recall” is not known until individual situations come into play. In other words, the values of the archived information cannot be perfectly predicted for later use when it is needed [46]. Thus, people often keep more than they need, and they do not expect to use all the information they have archived. At the same time, they still look for assistance with archiving or discarding decisions for better PHIM practices.

Finally, the findings suggest that 3 PIM Perception constructs entered in the last block are significantly associated with PHIM activity predictions. Accordingly, it appears that Awareness and Difficulty predict all PHIM activities, whereas Assistance only predicts the curatorial activities such as Labeling or Categorizing. On the basis of the findings, college students’ PHIM activities are influenced by their perceived behaviors, such as PIM awareness and difficulty. Relatedly, the Difficulty construct indicates the areas that information professionals, such as librarians and archivists, have traditionally addressed to assist their users. These professionals are trained to extract major informational values by anticipating future uses while discarding useless information from their public collections. Although archivists work for personal collections in their archives, their value-judgment is based on social, historical, and cultural schema. Although PHIM collections are archived for events of personal interest, information professionals’ skills and expertise can provide assistance in developing best PHIM practices, which could be taught to college students through training.

### Contribution

This paper contributed to understanding college students’ information management activities and tested multiple factors to predict major PHIM activities. In a large-scale survey, data collected from a young adult population with regards to personal record management is a novel finding in PIM studies. In particular, healthy young individuals in a college setting have been neglected in disease-based patient education as well as in health information–seeking studies. We believe that our findings provide critical values of information management skills among college students to promote self-help care management. Predictors that were tested range from demographic variation to academic measures to general PIM perceptions applied in a health information management setting. The findings about diverse PHIM activity constructs could be used as PHIM outcome measures for future validation, as in a training outcome measure for specific PHIM activity. We believe that further studies about cost-benefit analyses on diverse training methodologies on individual PHIM outcomes would even be beneficial to improve PHIM training design and evaluation. We also suggest validating diverse PHIM activity constructs with relevance to clinical or wellness outcomes.

### Limitations

This study presents a number of limitations. First, we used large-scale survey data locally collected from one state-owned university. Therefore, our sample might be biased when generalizing to other college settings. The demographics considered in this study represent a female-dominant sample. Although other demographic or academic variables, except for gender, were found to be insignificant predictors, it is advisable to use a more diverse ethnic group in future studies. Additionally, technological variations did not form any significant factor in our analyses. However, our results could change if we collect data years after PHRs or other patient portal services are introduced to the arena of college health. As of today, our participants were not fully aware of PHRs or other patient portal services that could be utilized for managing PHRs. Therefore, further postimplementation studies with PHR users are needed for validating some of our findings.

### Conclusions

In conclusion, PHIM among college students is a neglected topic of health information seeking, health services, or health IT research and tool development. Due to the relatively healthy nature of young adults in a rich educational environment, their information management skills could be improved drastically once we pay close attention to individual PHIM skill training and development. The findings of this study indicate that the awareness of PHIM’s importance is in place, but the reality of weak skills, such as curatorial activity and least utilized records such as patient education materials, should be acknowledged and remedied while in college or by hospitals’ health services. Most particularly, a special focus should be given to train college students about how to assess informational values of personal records and their efficient organization by utilizing various health information technologies. Public collections management and strategies devised for public use in libraries or archives may not be suitable for personal archival or health record collection management. The dynamic nature of informational values or individual levels of health information literacy will come to the forefront in a personalized PHIM education setting. With the advent of PHRs or any similar patient portal services by large academic hospitals or individual providers, college students will be faced with overloaded health records that have never been addressed in their education [47]. For preventive care, numerous studies have confirmed that informed health consumers exhibit better self-care than those that are not informed. As college students’ independence starts from their college years, their health record management is soon to be declared free from parental control. Understanding information flow and values of their personal records is important in understanding college students’ health conditions, and relevant PHIM educational endeavors will definitely boost these efforts.

## Abbreviations

PCA: principal component analysis
PHIM: personal health information management
PHRs: personal health records
PIM: personal information management

## Appendix

Multimedia Appendix 1 Primary factors in personal health information management (PHIM) activities for record types.

Multimedia Appendix 2 Aggregate result of hierarchical regression analyses for all the 12 personal health information management (PHIM) constructs.
