# Supplementary material for: Analysis of College Students’ Personal Health Information Activities: Online Survey
Source: J Med Internet Res. 2018 Apr 20;20(4):e132. doi: 10.2196/jmir.9391 (PMC5935801; doi:10.2196/jmir.9391)
Supplement: Multimedia Appendix 1 [file jmir_v20i4e132_app1.pdf]

Appendix 1.

Primary factors in personal health information management (PHIM) activities for record types.

Component		1	2	3	4	5	6	7	8	9	10	11
**Factor 1: Labeling**												
	Emergency	0.736										
	Surgery	0.711										
	Family medical history	0.710										
	Drugs	0.699										
	Insurance	0.687										
	Immunology	0.667										
**Factor 2: Sharing**												
	Emergency		0.720									
	Surgery		0.716									
	Family medical history		0.701									
	Drugs		0.687									
	Emergency		0.671									
**Factor 3: Categorizing**												
	Surgery			0.830								
	Family medical history			0.817								
	Drugs			0.817								
	Emergency			0.808								
	Insurance			0.806								

	Immunology			0.801								
	Education			0.749								
**Factor 4: Collecting**												
	Surgery				0.712							
	Drugs				0.692							
	Emergency				0.676							
	Family medical history				0.666							
**Factor 5: Health education materials**												
	Share					0.657						
	Collect					0.651						
	Use					0.644						
	Find					0.640						
	Own					0.638						
**Factor 6: Knowing**												
	Emergency						0.760					
	Surgery						0.757					
	Family medical history						0.757					
	Drugs						0.737					
	Insurance						0.664					
	Immunology						0.642					
**Factor 7: Discarding**												
	Surgery							0.925				

	Family medical history							0.919				
	Emergency							0.918				
	Immunology							0.893				
	Drugs							0.879				
	Insurance							0.812				
	Education							0.646				
**Factor 8: Insurance information**												
	Find								0.676			
	Share								0.640			
	Own								0.637			
**Factor 9: Organizing**												
	Surgery									0.703		
	Emergency									0.688		
	Family medical history									0.686		
	Drugs									0.677		
	Immunization									0.668		
**Factor 10: Using**												
	Family medical history										0.717	
	Emergency										0.714	
	Surgery										0.710	
	Insurance										0.678	

	Drugs										0.675	
**Factor 11: Owning**												
	Surgery											0.709
	Emergency											0.702
	Family medical history											0.696
	Drugs											0.694
	Immunology											0.673
Cronbach alpha		.969	.933	.971	.924	.895	.944	.925	.803	.964	.922	.933
Mean		3.052	3.550	2.500	3.335	2.962	3.795	2.414	3.933	3.250	3.692	3.252
Standard deviation		1.347	1.244	1.205	1.270	1.305	1.166	1.179	1.113	1.289	1.162	1.329
Eigenvalue		40.867	6.606	4.502	2.901	2.415	2.102	2.050	1.727	1.655	1.508	1.420
Percentage of variance explained		48.651	7.865	5.359	3.454	2.875	2.503	2.440	2.056	1.970	1.795	1.690
