# Supplementary material for: Analysis of College Students’ Personal Health Information Activities: Online Survey
Source: J Med Internet Res. 2018 Apr 20;20(4):e132. doi: 10.2196/jmir.9391 (PMC5935801; doi:10.2196/jmir.9391)
Supplement: Multimedia Appendix 2 [file jmir_v20i4e132_app2.pdf]

Appendix 2.

Aggregate result of hierarchical regression analyses for all the 12 personal health information management (PHIM) constructs.

Dependent predictors		Overall Personal Health	Lab	Sha	Categ	Collect	Health	Kno	Disca	Insurance	Orga	Us	Ow
Statistics		P/R^2	P/R^2	P/R^2	P/R^2	P/R^2	P/R^2	P/R^2	P/R^2	P/R^2	P/R^2	P/R^2	P/R^2
**Demographics**													
	Overall	.000[b]	.067	.000[b]	.624	.000[b]	.191	.000[b]	.015	.000[b]	.000[b]	.000[b]	.000[b]
	Age	.174	.623	.168	.613	.054	.447	.056	.310	.588	.010[b]	.190	.223
	Gender	.004[a]	.159	.005[a]	.779	.001[b]	.564	.000[b]	.293	.001[b]	.095	.001[b]	.000[b]
	Ethnicity	.345	.828	.105	.402	.331	.755	.086	.354	.012[a]	.548	.002[a]	.835
	Relationship	.164	.219	.636	.362	.766	.405	.426	.027	.358	.081	.819	.873

	R^2	0.019	0.008	0.020	0.002	0.019	0.006	0.034	0.012	0.033	0.020	0.030	0.022
**Academics**													
	Overall	.001[b]	.235	.001[b]	.367	.001[b]	.013	.000[b]	.014	.000[b]	.001[b]	.000[b]	.001[b]
	GPA	.279	.908	.975	.135	.286	.006[a]	.086	.382	.317	.281	.696	.622
	Number of courses taken	.755	.727	.643	.244	.524	.820	.656	.030	.717	.302	.895	.837
	Academic Status	.160	.244	.016[a]	.515	.045[a]	.069	.623	.719	.267	.302	.904	.901
	R^2	0.022	0.009	0.024	0.007	0.023	0.017	0.037	0.017	0.036	0.023	0.030	0.022
**Health and Information Resources**													
	Overall	.000[b]	.001[b]	.000[b]	.000[a]	.000[b]	.000[b]	.000[b]	.005[a]	.000[b]	.000[b]	.000[b]	.000[b]
	Number of clinic visit	.050[a]	.307	.006	.523	.051	.264	.150	.056	.052	.276	.00	.399

				[a]								3[a]	
	Professional	.001[b]	.054	.000[b]	.002[a]	.002[a]	.000[b]	.028[a]	.525	.534	.006[a]	.013	.039[a]
	Family	.657	.924	.086	.416	.463	.709	.765	.556	.047[a]	.965	.683	.959
	Colleague	.009[a]	.024[a]	.047[a]	.005[a]	.041[a]	.003[a]	.088	.275	.350	.024[a]	.592	.083
	Internet	.217	.565	.498	.411	.233	.821	.127	.772	.720	.037[a]	.242	.467
	Mass Media	.189	.147	.076	.656	.670	.688	.228	.179	.056	.393	.158	.015[a]
	R^2	0.069	0.033	0.067	0.043	0.061	0.074	0.067	0.029	0.056	0.061	0.064	0.053
**Personal information management perceptions**													
	Overall	.000[b]	.000[b]	.000[b]	.000[a]	.000[b]	.000[b]	.000[b]	.000[a]	.000[b]	.000[b]	.000[b]	.000[b]
	Assistance	.027[a]	.	.	.260	.351	.723	.149	.413	.432	.001[b]	.	.

			001[b]	027[a]								939	132
	Awareness	.000[b]	.000[b]	.000[b]	.000[b]	.000[b]	.000[b]	.000[b]	.000[a]	.000[b]	.000[b]	.000[b]	.000[b]
	Difficulty	.001[b]	.000[b]	.002[a]	.027[a]	.004[a]	.182	.001[b]	.181	.006[a]	.000[b]	.030[a]	.002[a]
	R^2	0.144	0.103	0.122	0.068	0.113	0.104	0.128	0.043	0.105	0.131	0.121	0.119

[a]P<.05.

[b]P<.001.
